# A phytochemical–nanotechnology platform exhibits complementary antimicrobial and immunomodulatory effects in experimental fowl typhoid

**DOI:** 10.3389/fmicb.2026.1894409

**Published:** 2026-10-01

**Authors:** Afnan Saleem, Baqia Farhat, Yaawar Mir, Tasaduq Manzoor, Junaid Nazir, Sheikh F. Ahmad, Abdul Majeed Ganai, Sabry M. Attia, Minakshi Prasad, Syed Mudasir Ahmad

**Affiliations:** 1 Watson-Crick Centre for Molecular Medicine, Islamic University of Science & Technology, Pulwama, India; 2 Division of Animal Biotechnology, FVSC & AH, Sher-e-Kashmir University of Agricultural Sciences & Technology, Srinagar, India; 3 Department of Zoology, University of Kashmir, Srinagar, India; 4 Department of Pharmaceutical Sciences, College of Pharmacy, Oregon State University, Corvallis, OR, United States; 5 Department of Pharmacology and Toxicology, College of Pharmacy, King Saud University, Riyadh, Saudi Arabia; 6 Division of Animal Nutrition, FVSC & AH, Sher-e-Kashmir University of Agricultural Sciences & Technology, Srinagar, India; 7 Lala Lajpat Rai University of Veterinary & Animal Sciences, Hisar, India

**Keywords:** 4-hydroxycoumarin, antimicrobial resistance, phytoremediation, poultry, *Salmonella gallinarum*

## Abstract

*Salmonella gallinarum*, the causative agent of fowl typhoid, poses a persistent threat to poultry health and productivity, compounded by the growing problem of antibiotic resistance. Addressing the urgent need for effective alternatives, this study evaluated a multi-component complementary approach combining the phytocompound 4-hydroxycoumarin, silver nanoparticles (AgNPs), and Ajwain (*Trachyspermum ammi*) extract. *In vitro* assays, including antimicrobial susceptibility testing, cytotoxicity, and concentration optimization, confirmed the potent antibacterial activity and favorable safety profiles of the tested components. Molecular docking identified a putative interaction between 4-hydroxycoumarin and Chemokine (C-C motif) Ligand 5 (CCL5). Analysis of the archived molecular dynamics trajectory, after correction for periodic boundary conditions, revealed substantial protein conformational deviation and increasing ligand displacement toward the end of the trajectory, with a loss of close protein–ligand contact after approximately 40 ns. These findings support a predicted interaction but do not establish persistent ligand binding or a single predicted protein–ligand association mode. In the chicken infection model, the combination treatments were associated with descriptively reduced clinical severity and differences in endpoint mortality. These *in vivo* findings should be regarded as preliminary, descriptive, and exploratory proof-of-concept data, as mortality and clinical endpoint comparisons did not remain statistically significant after Holm correction. As 4-hydroxycoumarin was evaluated *in vivo* only as a component of the combination formulations, the observed effects belong strictly to the tested combinations, and their individual contributions cannot be determined independently. Gene expression analyses further revealed a downregulation of proinflammatory cytokines, providing exploratory evidence of immunomodulatory responses associated with the tested formulations. Together, these findings provide preliminary proof of concept for a multi-component approach against *S. gallinarum* infection.

## Introduction

*Salmonella gallinarum* is a host-specific pathogen that primarily infects poultry, causing a severe systemic infection known as fowl typhoid, a septicaemic disease with high morbidity and mortality affecting chicks of all ages. It is a natural flagellate Salmonella Serovar that is highly adapted to poultry and has a limited host range (Ojima et al., 2022). Salmonella infections in poultry can occur through various routes, including the fecal–oral route, contaminated feed or water, and vertical transmission from infected hens to their offspring (Andino and Hanning, 2015). The disease can cause high mortality rates in infected birds, leading to significant financial losses for poultry farmers. Additionally, infected birds may experience reduced production, including decreased egg production and poor growth rates, further impacting the industry’s profitability (de Oliveira et al., 2005).

Common strategies to combat Salmonella infections include vaccination, good hygiene, and antimicrobial therapy; however, antibiotics are often preferred because immunization is not always practical or effective in certain contexts. Unlike vaccines, which require time to generate an immune response and primarily serve preventive purposes, antibiotics provide immediate therapeutic action by directly targeting and killing bacteria, thereby alleviating symptoms and preventing disease progression (Buckley et al., 2019). Despite these advantages, the widespread and indiscriminate use of antibiotics has led to two critical issues: the emergence of antibiotic-resistant microorganisms and the accumulation of antibiotic residues in food products intended for human consumption (Rodrigues et al., 2020). The World Health Organization (WHO) and CDC have recognized antimicrobial resistance (AMR) as one of the most pressing global health threats, predicted to cause up to 10 million annual deaths and an economic burden of USD 100 trillion by 2050 (Tang et al., 2017; Saha and Sarkar, 2021). The poultry industry, under increasing pressure from WHO and European Union (EU) regulations to reduce the use of antibiotics as feed additives and growth promoters, urgently requires effective and sustainable alternatives (Abd El-Hack et al., 2022). This concern is further exacerbated by the emergence of multidrug-resistant (MDR) Salmonella strains that resist multiple classes of antimicrobials, severely limiting treatment options and increasing the risk of therapeutic failure. MDR isolates have been reported in retail meats, poultry farms, and clinical samples worldwide, highlighting the public health risks associated with indiscriminate antibiotic use in poultry production (Tran et al., 2023).

Natural product–based compounds present a promising alternative to conventional antibiotics, as their diverse chemical structures make it difficult for bacteria to develop resistance. In organic poultry production, emphasis is placed on using such alternatives to both prevent disease and enhance overall bird performance. The health-promoting effects of plants and plant extracts are largely attributed to their ability to stimulate the production of endogenous digestive enzymes and boost antioxidant defenses (Lee et al., 2004). Unlike synthetic antibiotics or inorganic chemicals, plant-derived molecules are generally safer, less toxic, and free from harmful residues in animal products. Moreover, these natural compounds help maintain a balanced gut microbiome, reduce the production of microbial toxins, and alleviate inflammation, thereby supporting both animal welfare and sustainable poultry farming practices.

Coumarins represent a large family of naturally occurring and synthetic compounds widely recognized for their diverse pharmacological activities, including anticoagulant, anti-inflammatory, antioxidant, anticancer, and antimicrobial effects (Venugopala et al., 2013; Emami and Dadashpour, 2015). Among them, 4-hydroxycoumarin and its derivatives have drawn particular attention due to their broad-spectrum biological activities and relatively low toxicity profile. Several studies have highlighted the antibacterial activity of 4-hydroxycoumarin derivatives against Gram-positive and Gram-negative bacteria, with mechanisms linked to interference with bacterial cell wall synthesis, membrane disruption, and inhibition of key microbial enzymes (Bhakta et al., 2025; Sahoo et al., 2021). In addition, coumarins are reported to modulate quorum sensing, thereby reducing bacterial virulence and biofilm formation (Reen et al., 2018).

Despite existing evidence on the broad-spectrum antimicrobial activity of 4-hydroxycoumarin, its efficacy against *S. gallinarum*-the causative agent of fowl typhoid-has not yet been systematically investigated. Given the growing prevalence of multidrug-resistant Salmonella strains in poultry production, evaluating phytocompounds as potential therapeutic alternatives offers a promising strategy for sustainable disease management and reducing dependence on conventional antibiotics in the poultry industry. In this context, our study assessed the antimicrobial efficacy of the phytocompound 4-hydroxycoumarin in combination with Ajwain and silver nanoparticles against *S. gallinarum* infection. Silver nanoparticles may potentially enhance the stability and delivery of bioactive compounds, as reported previously. Furthermore, we examined the treatment’s influence on pathogenesis-related gene expression to gain comprehensive insights into its antibacterial potential and systemic effects.

## Materials and methods

### Bacterial strain and culture conditions

*Salmonella enterica* Serovar Gallinarum (ATCC 9184) was incubated in Tetrathionate broth (TTB) (Himedia) for 48 h at 42 °C. The incubated culture was examined for Salmonella colonies after streaking on Salmonella-Shigella (SS) and MacConkey agar, and on Xylose-Lysine-Deoxycholate (XLD) agar (Himedia).

Colony-forming units were determined by counting the colonies on SS agar plates from each dilution after overnight incubation.


$$\begin{array}{l} (\text{Number of Colonies Counted}\times \text{Dilution Factor})/\text{Aliquot Volume}\\ =\text{Colony Forming Unit}\;(\mathrm{CFU}) \end{array}$$

### Phytochemical synthesis

A stirred suspension of sodium hydride (60% dispersion in mineral oil; Sigma-Aldrich, St. Louis, MO, USA) (60 mmol) was prepared in anhydrous toluene (Merck, Darmstadt, Germany) (30 mL). A solution of 2-hydroxyacetophenone (Sigma-Aldrich, St. Louis, MO, USA) (1.2 mmol) and the acylating reagent (Sigma-Aldrich, St. Louis, MO, USA) (1.8 mmol) dissolved in anhydrous toluene (6 mL) was added dropwise at room temperature. After the addition was complete, the reaction mixture was heated at 100 °C for 3 h. The reaction mixture was then cooled to room temperature, the solvent was evaporated under reduced pressure, and the residue was treated with distilled water (10 mL). The resulting precipitate was filtered, washed thoroughly with distilled water, and recrystallized from methanol (Merck, Darmstadt, Germany) or ethanol (Merck, Darmstadt, Germany) to obtain 4-hydroxycoumarin. The synthesized compound was characterized by mass spectrometry, Fourier transform infrared (FTIR) spectroscopy, and ^1^H and carbon-13 (C13) nuclear magnetic resonance (^13^C NMR) spectroscopy. The purity of 4-hydroxycoumarin was determined by reverse-phase high-performance liquid chromatography (HPLC) using detection wavelengths of 254 and 210 nm.

Ajwain is a medicinal plant belonging to the Apiaceae family and possesses broad-spectrum antimicrobial activity and was isolated using a previously described protocol (Haq et al., 2022). Seeds were obtained and dried at room temperature (30 ± 2 °C) for 3 days. Dried seeds were then ground into a fine powder and sieved through a sieve (aperture size: 0.3 mm). The powder was then subjected to successive extraction with 95% ethanol (Merck, Darmstadt, Germany) in a Soxhlet extractor (60–80 °C). The ethanolic extract was also concentrated in a rotary evaporator under reduced pressure. The extract was stored in a refrigerator at 4 °C until further use.

### Nanoparticle synthesis and characterization

Silver nanoparticles were synthesized using garlic extract as the reducing agent (Gregory et al., 2012). Fresh garlic bulbs were procured from a local certified vegetable market in Srinagar, Jammu and Kashmir, India. Garlic was peeled and rinsed with deionized water. Garlic was then allowed to dry in the open air to remove any remaining moisture, and was finely ground using an autoclaved mortar and pestle. Ground garlic paste (9.8 g) was mixed with deionized water (25 mL). The mixture was placed on a magnetic stirrer with a hot plate and boiled at 100 °C for 30 min, followed by centrifugation at 4,000 rpm for 30 min. Using a Whatman filter paper, the supernatant was filtered, and the extract was stored at 4 °C. Garlic extract solution volumes (ranging from 1.0–2.5 mL) were introduced into a 0.98 mM AgNO_3_ solution (Sigma-Aldrich, USA) (51 mL dissolved in deionized water). A change in color to light orange within 2 h indicates the formation of silver nanoparticles. After 48 h, the solution matures to a deep orange/brown color. Silver nanoparticle dispersions were centrifuged for 15 min at 8,000 rpm to eliminate any large aggregates and excess garlic extract. The yellow/orange supernatant was collected and retained as the final silver nanoparticle product, while the precipitate was discarded.

Characterization of silver nanoparticles was performed using scanning electron microscopy (SEM) to evaluate their surface morphology, particle shape, and approximate nanoscale dimensions. The synthesized garlic-mediated AgNPs were further analyzed by FTIR to identify the major functional groups on the nanoparticle surface. The FTIR spectrum was recorded over the relevant wavenumber range, and the characteristic absorption bands were used to assess the involvement of phytochemical functional groups in nanoparticle synthesis and stabilization.

### Disk diffusion test

The antibacterial activity of the phytochemicals was assessed by a disk diffusion test. Mueller-Hinton agar plates were uniformly inoculated with approximately 2 × 10^8^ CFU/mL of *S. gallinarum* using sterile cotton swabs. Sterile 6-mm filter paper disks impregnated with the respective phytocompounds or with ciprofloxacin were placed on the agar surface and incubated at 37 °C for 24 h.

The diameter of the inhibition zone (IZ) around the disks was measured to evaluate the antibacterial activity. The zone of inhibition of the antibiotic disk (ciprofloxacin disk) was compared to the zone of inhibition around the phytochemical-impregnated disk to determine the susceptibility of the bacteria to the phytochemicals and nanoparticles. The disk-diffusion assay was used as a preliminary qualitative screening method to assess antibacterial activity. As the tested agents differed substantially in the amount loaded per disk and in their physicochemical properties and diffusion behavior, inhibition-zone diameters were not considered directly equivalent to the activity of ciprofloxacin.

### Minimum inhibitory concentration (MIC) and minimum bactericidal concentration (MBC) assays

MICs were determined by the broth microdilution method in 96-well plates. Serial dilutions of 4-hydroxycoumarin, AgNPs, and Ajwain extract were prepared in Mueller-Hinton broth, noculated with *Salmonella gallinarum* (5 × 10^5^ CFU/mL), and incubated at 37 °C for 18–24 h. MIC was determined as the lowest concentration at which no visible bacterial growth was observed. MBC was determined by subculturing aliquots from growth-inhibited wells onto Mueller-Hinton agar, and the lowest concentration that showed no colony growth after 24 h was recorded as the MBC. All the assays were performed in triplicate.

### Cytotoxicity screening

Cytotoxicity of the selected phytocompounds was evaluated using the 3-(4,5-dimethylthiazol-2-yl)-2,5-diphenyltetrazolium bromide (MTT) assay (Sigma-Aldrich, St. Louis, MO, USA). Chicken fibroblast cells, kindly provided by Dr. Nadeem Shabir, were cultured in Dulbecco’s Modified Eagle Medium (DMEM; Gibco, Thermo Fisher Scientific, USA) supplemented with 10% fetal bovine serum (FBS; Gibco, Thermo Fisher Scientific, USA) and 1% penicillin–streptomycin (Gibco, Thermo Fisher Scientific, USA). Cells were maintained at 37 °C in a humidified incubator with 5% CO₂. Approximately 1 × 10^4^ cells per well were seeded into 96-well plates and allowed to reach approximately 80% confluence before treatment. At 80% confluency, the medium was replaced with medium containing different concentrations of selected phytocompounds (4-hydroxycoumarin: 20 mg/mL, Ajwain: 30 mg/mL, and silver nanoparticles: 100 μg/mL, ciprofloxacin: 5 μg/disk). These concentrations were selected based on the published literature, preliminary optimization experiments, and cytotoxicity assessment. After a 24-h incubation period, MTT solution (5 mg/mL) was added, and the cells were incubated for 4 h. 100 μL of dimethyl sulfoxide (DMSO, Sigma-Aldrich, USA) solvent was added in each well after incubation, and the optical density of the formazan product was read in a microplate reader (570 nm).

### Docking analysis

#### Molecular docking

Molecular docking was performed using the crystal structure of human Chemokine (C-C motif) Ligand 5 (CCL5) (PDL5 ID: 1EQT), which was prepared by adding hydrogens, removing water molecules, heteroatoms, and ligands, and converting to modified Protein Data Bank partial charges (Q) and AutoDock atom types (T) (PDBQT) format with Gasteiger charges. In contrast, the ligand 4-hydroxycoumarin was optimized and similarly formatted. Using the cavity analysis tool, a putative ligand-binding cavity was identified and used for docking. AutoDock Vina was employed for docking using a rigid-receptor, flexible-ligand approach, generating nine poses ranked by binding affinity. Cross-docking using Smina and GNINA was also conducted to validate the results using rescoring and deep learning-based affinity evaluation. Based on the consensus binding orientation and favorable docking scores, the top-ranked pose, with a predicted binding score of −8.3 kcal/mol, was selected for subsequent molecular dynamics simulation.

#### Homology modeling and structural validation of chicken CCL5

To further evaluate the suitability of the computational target for the avian infection model, we retrieved the chicken CCL5 amino acid sequence from the UniProt database (Accession number: Q8QG57) and compared it with experimentally resolved human CCL5 (Protein Data Bank [PDB] ID: 1EQT). Pairwise sequence alignment was performed to assess the sequence conservation between the two orthologs. Three-dimensional (3D) structure of chicken CCL5 was obtained from the AlphaFold Protein Structure Database and structurally superposed onto the human CCL5 crystal structure using PyMOL.

Structural similarity was assessed by backbone root-mean-square deviation (RMSD). Confidence in the AlphaFold-predicted structure was assessed using per-residue predicted Local Distance Difference Test (pLDDT) scores to evaluate the reliability of the mature chemokine domain.

The structural conservation was further validated using MODELLER 10.8. Using a high-resolution human CCL5 crystal structure (PDB ID: 5COY) as the template, an independent comparative homology model of the mature chicken CCL5 was generated using MODELLER. 20 independent models were generated and ranked according to the GA341 quality and discrete optimized protein energy (DOPE) score. The best-scoring model was selected for structural comparison with the AlphaFold model. The final model was evaluated by structural superposition and comparison of the proposed ligand-binding region. These analyses were performed to assess the suitability of the avian CCL5 model for subsequent molecular docking studies.

#### Molecular dynamics trajectory

A 50 ns molecular dynamics simulation of the human CCL5–4-hydroxycoumarin complex was analyzed to evaluate protein structural stability and protein–ligand association. The trajectory was evaluated over the 5–50 ns interval. The interval from 20.002 to 21.800 ns was unavailable and was retained as an explicit data gap without reconstruction or interpolation.

Periodic boundary-condition artifacts were corrected prior to structural analysis, followed by reconstruction and structural fitting of the CCL5 dimer. Protein structural stability was assessed using Cα root-mean-square deviation (RMSD) for the reconstructed dimer and individual chains. Ligand RMSD was calculated following protein fitting to assess ligand positional displacement independently of overall protein motion. Protein–ligand association was evaluated using the minimum heavy-atom distance and the center-of-mass (COM) distance between the protein and the ligand. A distance of 0.45 nm was used as the threshold for close protein–ligand contact. These analyses were used to assess the structural stability of the CCL5 dimer and the temporal behavior of the 4-hydroxycoumarin–CCL5 association during the molecular dynamics trajectory.

#### Experimental design and Salmonella challenge

A total of 36 one-day-old Cobb broiler chicks were housed under standard brooding conditions at the Faculty of Veterinary Sciences and Animal Husbandry and provided a basal diet with ad libitum access to feed and water. Following a 3-day acclimatization period, the chicks were randomly assigned into six experimental groups (*n* = 6 birds per group) to minimize selection bias. The experiment was designed as an exploratory proof-of-concept study, with six birds assigned to each experimental group. A formal prospective sample-size or statistical power calculation was not performed before initiation of the study. Therefore, the study was intended to generate preliminary comparative data on clinical manifestations, mortality, pathological findings, and inflammatory gene expression responses rather than to provide definitive evidence of differential treatment efficacy between groups.

On day 4, chicks in the challenge groups were inoculated intraperitoneally with a sublethal dose of *S. gallinarum* (2 × 10^8^ CFU/mL). In contrast, the unchallenged control (CON) group received 1 mL of sterile nutrient broth orally as a mock challenge. Approximately 12 h post-infection, fecal swabs were collected and enriched overnight in tetrathionate broth at 42 °C, followed by streaking onto Salmonella–Shigella (SS) agar plates to confirm *Salmonella* infection.

Following confirmation of infection, the birds remained in their respective treatment groups and received the designated treatments for 5 consecutive days (days 6–10) while continuing on the basal diet. The six experimental groups were as follows: (i) unchallenged control (CON) (not experimentally infected with *S. gallinarum*); (ii) infected untreated (INF); (iii) infected birds treated with ciprofloxacin (ANT; 200 mg/100 kg body weight); (iv) infected birds treated with Ajwain (*T. ammi*) extract (AJ; 200 mg/kg body weight); (v) infected birds treated with Combination 1 (COM1; 4-hydroxycoumarin, 100 mg/kg body weight, plus silver nanoparticles, 50 mg/kg body weight); and (vi) infected birds treated with Combination 2 (COM2; Ajwain (*T. ammi*) extract, 200 mg/kg body weight, 4-hydroxycoumarin, 100 mg/kg body weight, and silver nanoparticles, 50 mg/kg body weight).

#### Sample collection

Chicks were euthanized with CO_2_ on day 11 of the experiment. A comprehensive necropsy examination was performed on the sacrificed bird’s carcass to document the typical Salmonellosis lesions. Bronze discoloration of the liver, vent paste, ruffled feathers, splenomegaly, and intestinal hemorrhages were observed. Samples were collected from the infected birds. For gross pathologies, the liver, spleen, heart, and digestive system were collected. For RNA isolation and downstream gene expression analysis, liver samples were collected.

#### Differential gene expression study

Trizol (Ambion, Thermo Fisher Scientific, Cat No: 15596018) was used to isolate RNA from tissue as per the manufacturer’s instructions. The quality of isolated RNA was checked using a Nanodrop (Thermo Scientific, USA). The samples showing an OD260/280 ratio between 1.9 and 2 were used for further experiments. cDNA synthesis (2 μg/μL of RNA/sample) was done with Revert Aid First Strand cDNA synthesis kit (Thermo Scientific, USA) following the manufacturer’s methods. The details of the primers used are given in the Supplementary file.

#### Real-time PCR quantification

Real-time PCR (RocheTM, Germany) was used to quantify mRNA expression levels of interleukin-6 (IL-6), IL-8, IL-12, IL-17A, inducible nitric oxide synthase (iNOS), and matrix metallopeptidase 1 (MMP1). Glyceraldehyde 3-phosphate dehydrogenase (GAPDH) and β-actin were used as internal controls. Denaturation at 95 °C for 5 min, annealing at variable temperatures (specific for each gene as shown in the Supplementary file) for 15 s, and extension at 72 °C for 15 s were the reaction conditions. Experiments were carried out in replicates. Using the 2^-∆∆CT^ method, data were presented as fold change (or fold difference) in target gene expression, normalized to endogenous reference gene relative to calibrator (Livak and Schmittgen, 2001). Unpaired Student′s *t*-test, one-way analysis of variance (ANOVA) with significance value set at *p* < 0.05 was used for statistical analysis.

## Results

### Spectroscopic characterization of 4-hydroxycoumarin

4-hydroxycoumarin was obtained as a pale-yellow crystal with a melting point of 210 °C.

Analytical data for NMR are given below (Figure 1).

**Figure 1:**
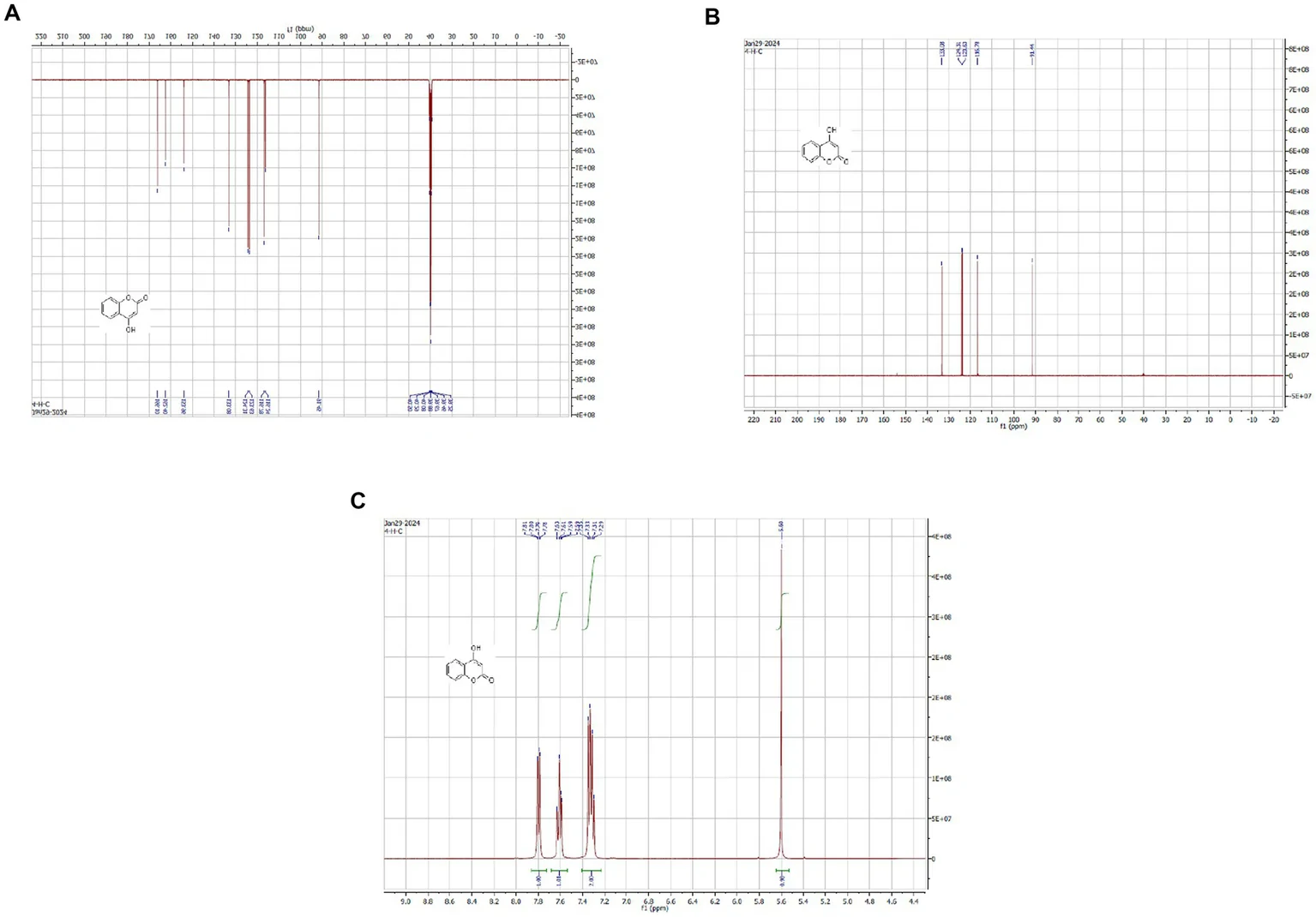
**(A)** ^1^H NMR spectrum of synthesized 4-hydroxycoumarin recorded in DMSO-*d*₆. The spectrum shows characteristic proton resonances corresponding to the aromatic and hydroxyl protons of 4-hydroxycoumarin, confirming successful synthesis and structural identity. **(B)** ^13^C NMR spectrum of synthesized 4-hydroxycoumarin recorded in DMSO-*d*₆. The spectrum exhibits the characteristic carbon resonances of the coumarin scaffold, including signals corresponding to the carbonyl, aromatic, and oxygenated carbon atoms, confirming the expected chemical structure and successful synthesis of 4-hydroxycoumarin. **(C)** Expanded view of the aromatic region (δ 6.5–8.0 ppm) of the ^1^H NMR spectrum of synthesized 4-hydroxycoumarin recorded in DMSO-*d*₆. The expanded spectrum clearly resolves the multiplet signals corresponding to the aromatic protons of the coumarin ring, facilitating peak assignment and confirming the expected proton environment and structural integrity of the synthesized 4-hydroxycoumarin.

IR (λ max, KBr) 3,387–2,583, 1,658, 1,599, 1,323 cm^−1^.

The FTIR spectrum exhibited a broad absorption band at 3,387–2,583 cm^−1^ corresponding to the hydrogen-bonded hydroxyl group. The strong band at 1,658 cm^−1^ confirmed the presence of the lactone carbonyl group characteristic of the coumarin nucleus. Additional peaks at 1,599 cm^−1^ and 1,323 cm^−1^ were consistent with aromatic C=C stretching and C–O vibrations, respectively.

^1^H NMR (400 MHz, DMSO) δ 7.80 (dd, *J* = 7.8, 1.0 Hz, ^1^H), 7.65–7.57 (m, ^1^H), 7.32 (dd, *J* = 13.8, 7.6 Hz, ^2^H), 5.60 (s, ^1^H).

^13^C NMR (101 MHz, DMSO) δ 166.10, 162.40, 153.96, 133.08, 124.31, 123.63, 116.78, 116.24, 91.4.

The successful synthesis of the target compound was confirmed by the ^1^H and ^13^C NMR spectra, which were consistent with the expected structure of 4-hydroxycoumarin and agreed well with previously reported spectral data.

MS (m/e) 162 (Mþ).

To confirm its identity, mass spectrometry was performed. It showed a molecular ion peak at m/z 162, corresponding to the molecular weight of 4-hydroxycoumarin.

HPLC analysis confirmed the high purity of the synthesized compound. HPLC analysis at 254 nm, the characteristic absorbance of the coumarin chromophore, showed a single dominant peak at 15.274 min accounting for 98.26% of the total area. Minor impurities were detectable at 210 nm, indicating minimal side-products from the synthesis (Figure 2).

**Figure 2:**
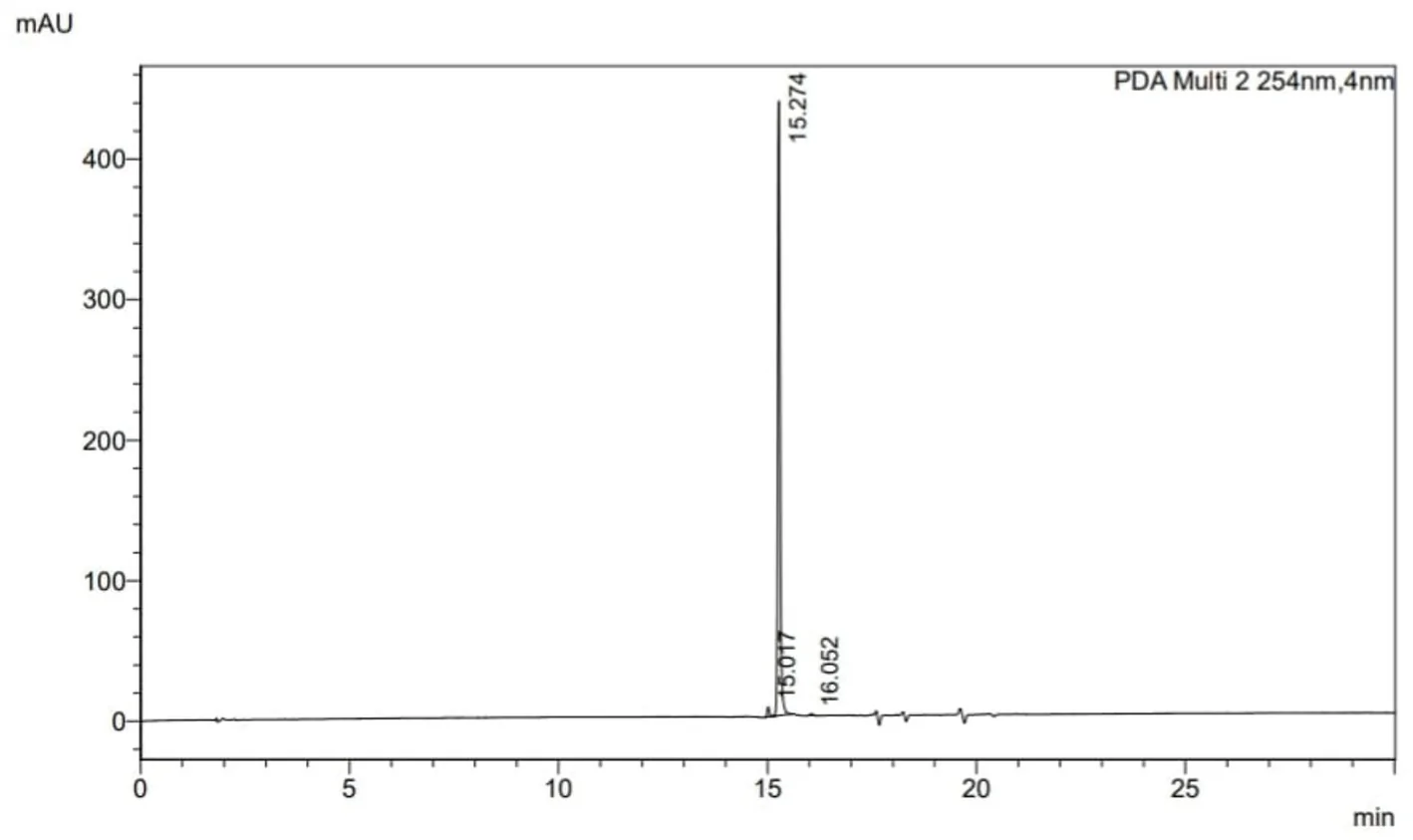
High-performance liquid chromatography (HPLC) chromatogram of synthesized 4-hydroxycoumarin recorded at 254 nm. The chromatogram was obtained using a reverse-phase HPLC system equipped with a photodiode array (PDA) detector. A single dominant peak was observed at a retention time of 15.274 min, accounting for 98.26% of the total peak area, confirming the high purity of the synthesized 4-hydroxycoumarin. The absence of significant additional peaks at 254 nm indicates minimal impurities, demonstrating the successful synthesis and purification of the target compound prior to its biological evaluation.

### Nanoparticle synthesis and characterization

The change in the solution’s color from pale gray to yellow and ultimately to brown indicated the progression of nanoparticle synthesis. Scanning electron microscopy revealed predominantly quasi-spherical particles with moderate agglomeration and nanoscale dimensions (Figure 3a). These observations provide morphological evidence for the formation of nanoparticle-like structures. FTIR analysis was performed to identify the functional groups associated with the reduction and stabilization of the synthesized silver nanoparticles (AgNPs) (Figure 3b). The spectrum displayed a broad absorption band at approximately ~3,300 cm^−1^, which may be attributed to O–H and/or N–H stretching vibrations, indicating the presence of hydroxyl- and amine-containing biomolecules associated with the nanoparticle surface. Additional absorption features were observed in the ~2,900 cm^−1^ region, followed by bands in the ~1,650–1,400 cm^−1^ range that may correspond to carbonyl, amide, C–H bending, and/or carboxylate-associated vibrations. An absorption feature in the ~1,100–1,200 cm^−1^ region was consistent with C–O or C–O–C stretching vibrations, suggesting the association of oxygen-containing plant-derived compounds with the synthesized nanoparticles. A prominent absorption band was also observed in the low-wavenumber region around ~550–600 cm^−1^. This feature may be associated with Ag–O-related vibrational contributions; however, its assignment should be considered tentative in the absence of complementary elemental and structural analyses. Collectively, the FTIR profile suggests that garlic-derived biomolecules participated in the reduction of Ag^+^ ions and the subsequent stabilization or capping of the synthesized AgNPs. Therefore, the present characterization should be interpreted primarily as morphological evidence. Further characterization using techniques such as energy-dispersive X-ray spectroscopy (EDX), X-ray diffraction (XRD), dynamic light scattering (DLS), and zeta-potential analysis will be required to establish nanoparticle composition, crystallinity, hydrodynamic size distribution, and colloidal properties (Table 1).

**Figure 3:**
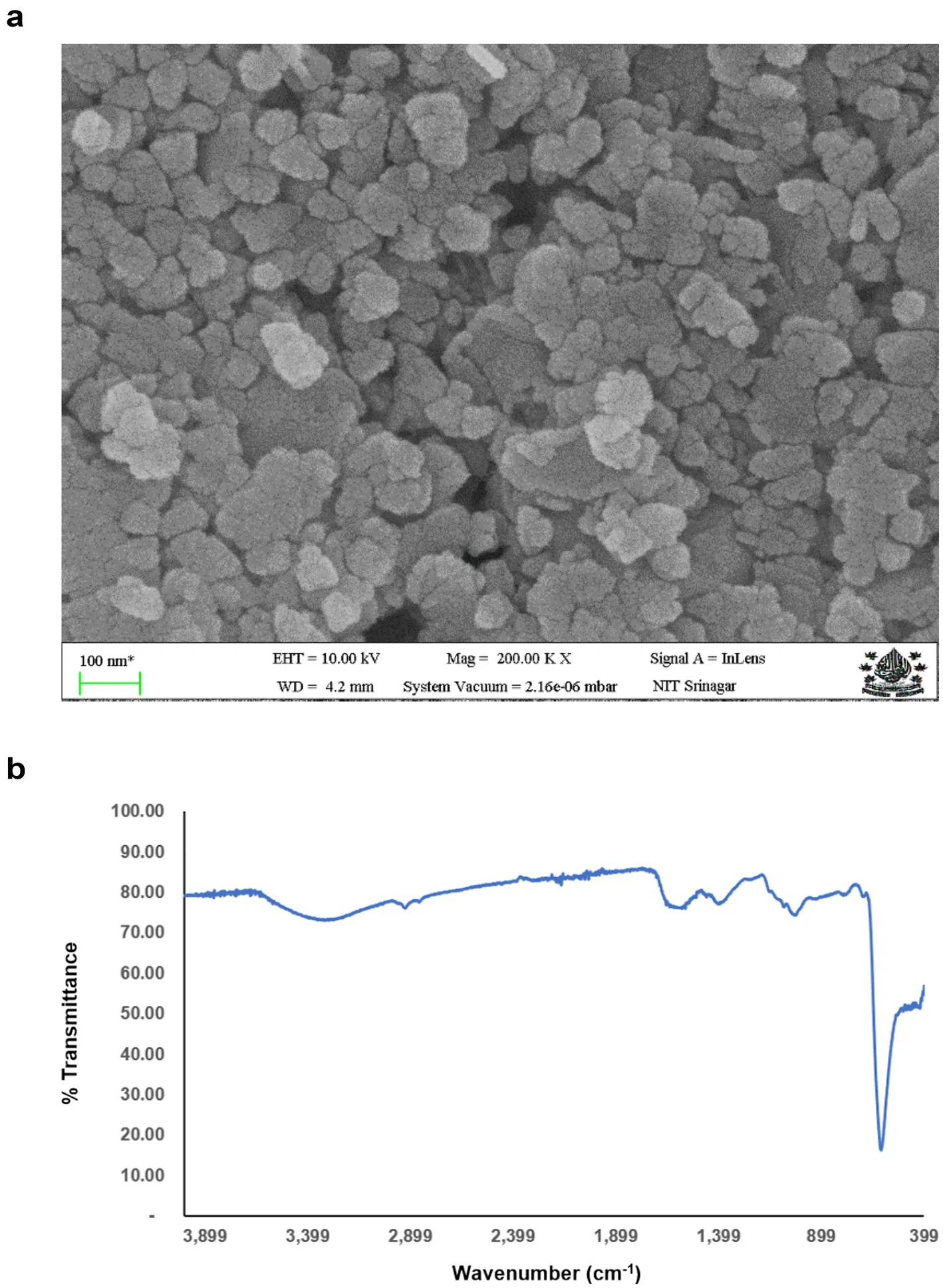
**(a)** Representative SEM image of silver nanoparticles using garlic extract. Scanning electron microscopy (SEM) image of the synthesized silver nanoparticles showing predominantly quasi-spherical nanoparticles with moderate agglomeration. The nanoparticles exhibit a relatively uniform morphology with nanoscale dimensions. Scale bar = 100 nm. **(b)** FTIR spectrum of garlic-mediated synthesized silver nanoparticles (AgNPs), showing absorption bands associated with surface-bound biomolecules potentially involved in the reduction and stabilization of the nanoparticles.

**Table 1:** Minimum inhibitory concentration (MIC), minimum bactericidal concentration (MBC), and MBC/MIC ratios of 4-hydroxycoumarin, garlic-synthesized silver nanoparticles (AgNPs), Ajwain extract, and ciprofloxacin against *Salmonella Gallinarum*.

Test agent	MIC (μg/mL)	MBC (μg/mL)	MBC/MIC
4-Hydroxycoumarin	256	>512	>2
AgNPs	16	32	2
Ajwain extract	128	256	2
Ciprofloxacin	0.25	0.5	2

### Disk diffusion test

The disk-diffusion assay provided preliminary evidence of antibacterial activity for the tested agents and was subsequently followed by MIC/MBC determination to quantitatively assess antibacterial potency. Disk diffusion screening demonstrated antibacterial properties of 4-hydroxycoumarin (20 mg/mL), Ajwain (30 mg/mL), and nanoparticles against *S. gallinarum.* Zones of inhibition were measured to assess the antibacterial activity. In our study, we found that the combination treatment exhibited a larger inhibition zone than its individual components (Supplementary Tables S2, S3). Thus, a combination treatment regimen was followed for *in vivo* trials in chicks. Phytocompounds that showed a greater inhibition zone against *S. gallinarum* are shown in Supplementary Figure S1.

The inhibition-zone measurements should be interpreted as preliminary screening data because the agents were tested at different mass loadings and their diffusion through agar may differ substantially. Direct comparison of inhibition-zone diameters with ciprofloxacin therefore does not constitute a comparison of antibacterial potency.

### MIC and MBC assays

MIC and MBC assays of the individual test agents were performed against *S. gallinarum* to evaluate their antibacterial potency. All test agents demonstrated antibacterial activity, although differences in inhibitory concentrations were observed among the individual compounds (Table 1).

### Cytotoxicity screening

The cytotoxic effect of extracted phytochemicals against the chicken fibroblast cell line was investigated using the MTT assay. Mean cell viability after 24 h of phytocompound treatment is represented in Figure 4. Mean cell viability showed a dose-dependent trend, indicating no substantial cytotoxic effect on cells. This suggests the use of these phytocompounds for *in vivo* trials on chicks.

**Figure 4:**
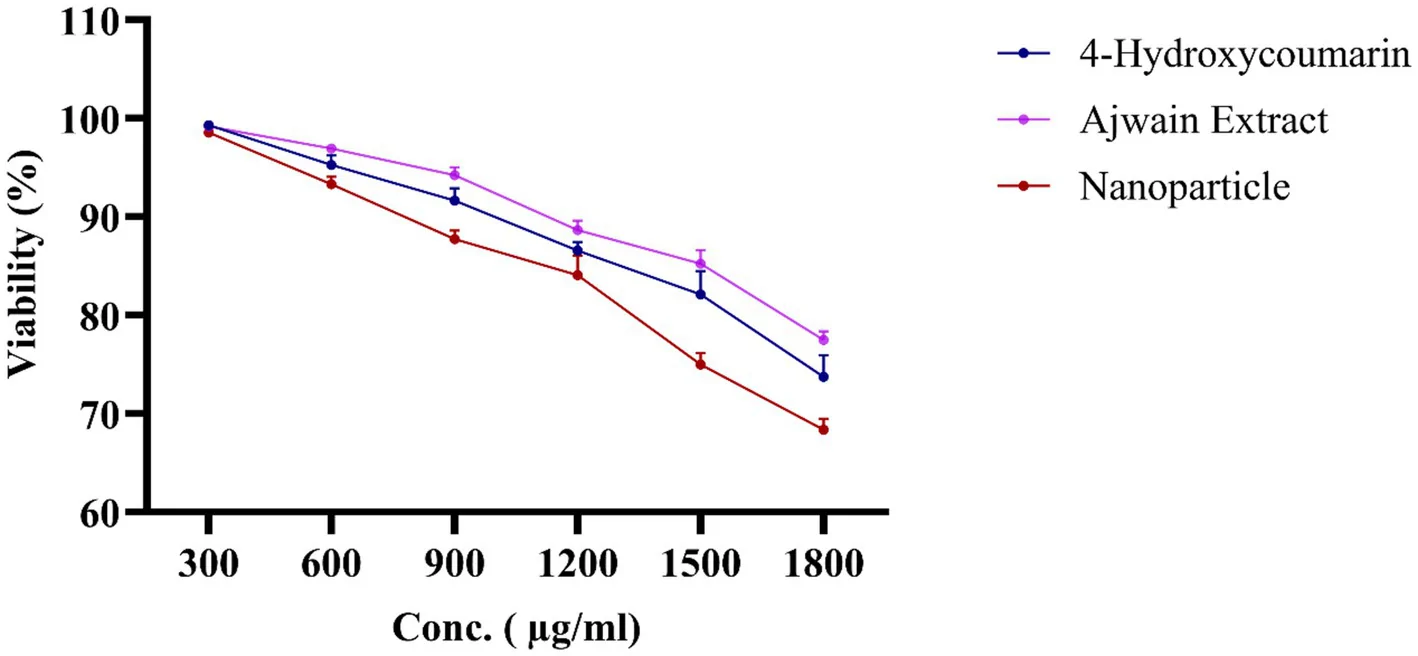
Effect of different phytocompounds on the viability of chicken fibroblast cells after 24 h of treatment. Cell viability was assessed using the 3-(4,5-dimethylthiazol-2-yl)-2,5-diphenyltetrazolium bromide (MTT) assay after 24 h of exposure to increasing concentrations of the tested phytocompounds. Cell viability is expressed as a percentage relative to the untreated control. The results demonstrate a concentration-dependent response, with the selected treatment concentrations showing no significant cytotoxic effects on chicken fibroblast cells. Data are presented as mean ± SEM from experiments performed in triplicate. Statistical analysis was performed using one-way ANOVA followed by Tukey’s multiple comparisons test, with statistical significance set at *p* < 0.05.

### Docking analysis

#### Molecular docking

CCL5 (Chemokine (C-C motif) ligand 5; Regulated on Activation, Normal T-cell Expressed and Secreted [RANTES]) was selected as the molecular target for computational analysis as it is a key chemokine involved in leukocyte recruitment and inflammatory signaling. Molecular docking analysis using AutoDock Vina identified a favorable predicted interaction between CCL5 and 4-hydroxycoumarin, with a best docking score of −6.3 kcal/mol. The predicted pose involved polar and non-polar contacts with residues surrounding a computationally identified ligand-accessible cavity. Cross-docking using Smina and GNINA provided additional computational support for the predicted interaction. As the site has not been experimentally established as a drug-binding pocket, these results are interpreted as a putative ligand-binding interaction rather than evidence of experimentally confirmed CCL5 binding. Cross-docking with Smina and GNINA robustly validated the results, confirming the consistency of the binding poses and energy. Based on the concordant docking poses obtained using multiple computational approaches, the top-ranked complex was selected for subsequent molecular dynamics simulation (Figure 5).

**Figure 5:**
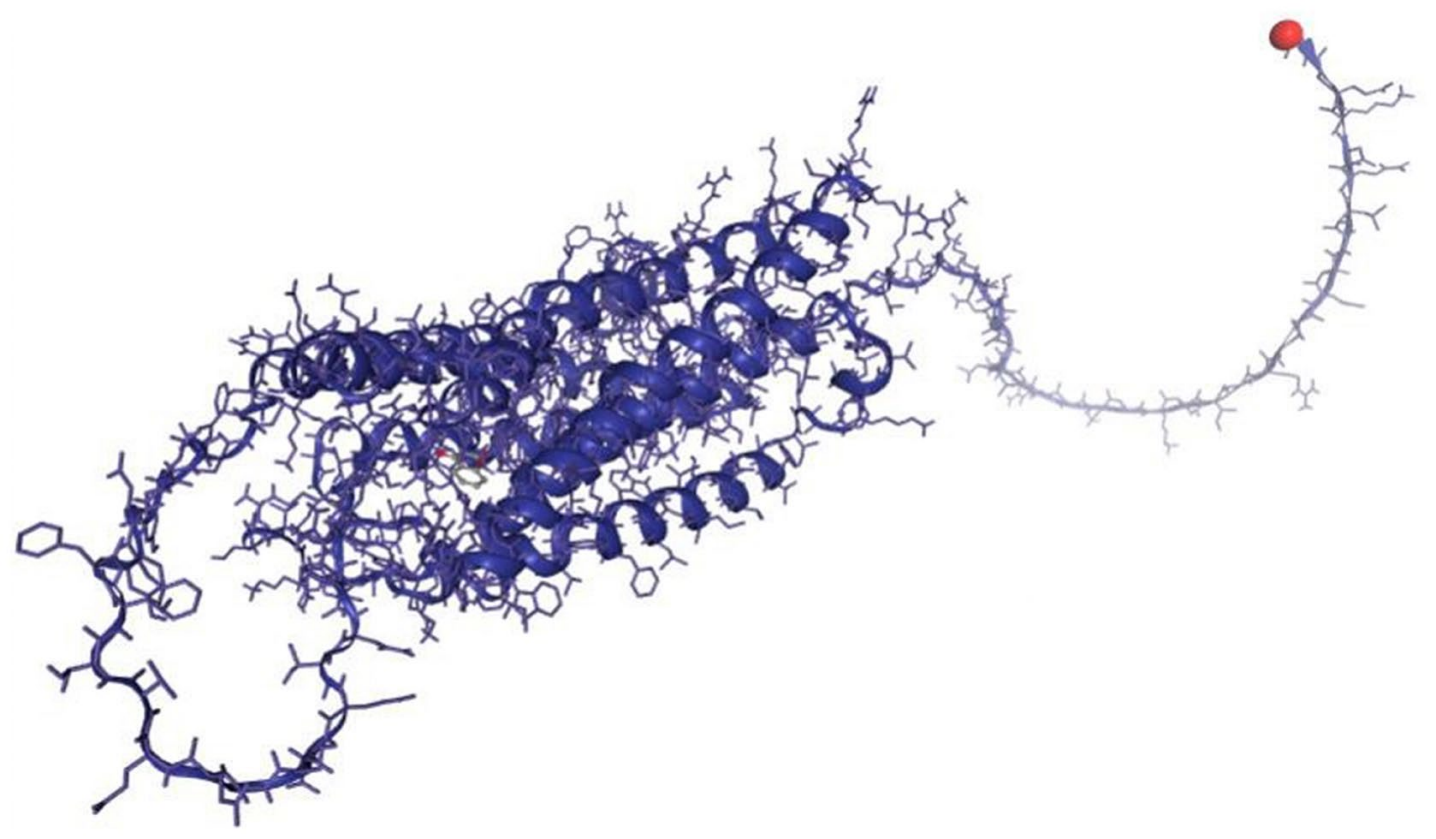
Molecular docking analysis of 4-hydroxycoumarin with human CCL5. The predicted ligand pose occupies a computationally identified ligand-accessible cavity and forms predicted polar and non-polar interactions with surrounding residues. The docking result represents a putative ligand-binding interaction and does not establish an experimentally validated CCL5 drug-binding site or biological inhibition.

#### Structural validation of the chicken CCL5 model

Pairwise sequence alignment between the two orthologs, that is, chicken CCL5 and human CCL5, demonstrated substantial evolutionary conservation. Both proteins exhibited 56% sequence identity and 70.3% sequence similarity, comprising 91 amino acid residues aligned without insertions or deletions. The conserved cysteine residues characteristic of the CC chemokine family, were retained in both proteins, indicating toward preservation of the canonical chemokine fold.

The AlphaFold-predicted chicken CCL5 model was structurally superpositioned on the experimentally determined human CCL5 crystal structure, demonstrating excellent structural agreement with a backbone RMSD of 0.42 Å. The overall architecture of the mature chemokine domain was maintained despite sequence divergence, and the characteristic α-helical region and β-sheet core were highly conserved. The structural reliability of the predicted model used for molecular docking was confirmed by analysis of the AlphaFold confidence map, which showed high pLDDT scores throughout the mature chemokine domain (Figure 6).

**Figure 6:**
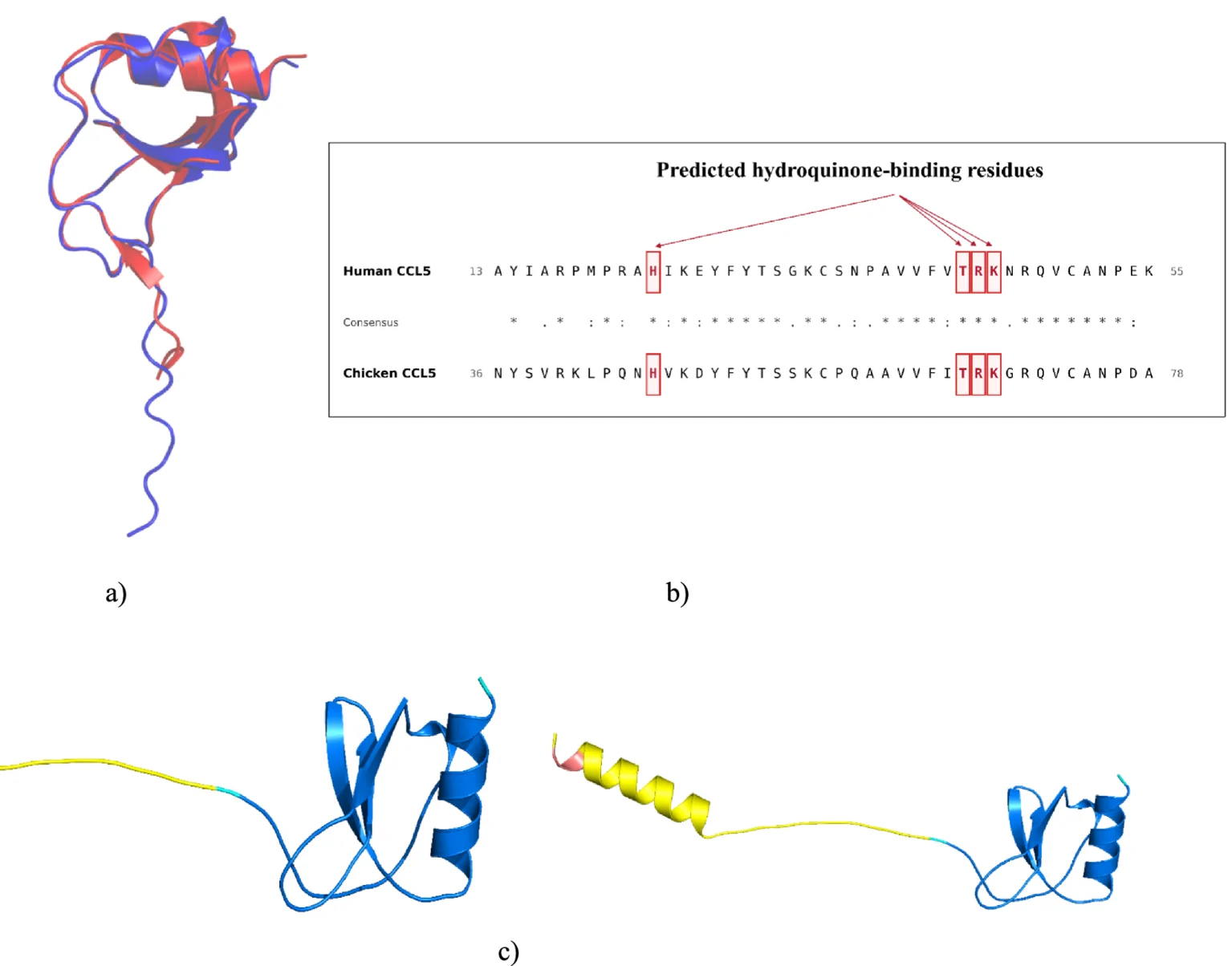
**(a)** Structural superposition of the AlphaFold-predicted chicken CCL5 (cyan) and the human CCL5 crystal structure (PDB ID: 1EQT; salmon). The mature chemokine domains exhibit excellent structural agreement with a backbone RMSD of 0.420 Å, demonstrating strong conservation of the overall chemokine fold; **(b)** Pairwise sequence alignment of human and chicken CCL5 highlighting conservation of residues forming the predicted hydroquinone-binding pocket; **(c)** AlphaFold confidence map of chicken CCL5. Dark blue, cyan, yellow, and orange represent pLDDT confidence scores of >90, 70–90, 50–70, and <50, respectively. High confidence across the mature chemokine domain supports the structural reliability of the AlphaFold model.

Further validating these observations, an independent comparative homology model of chicken CCL5 was generated using MODELLER (Figure 7). Structural comparison between the homology model and the AlphaFold structure demonstrated preservation of the overall chemokine fold and close agreement within the predicted ligand-binding region. Taken together, these findings support the structural suitability of the chicken CCL5 model for investigating the interaction of 4-hydroxycoumarin with the avian chemokine.

**Figure 7:**
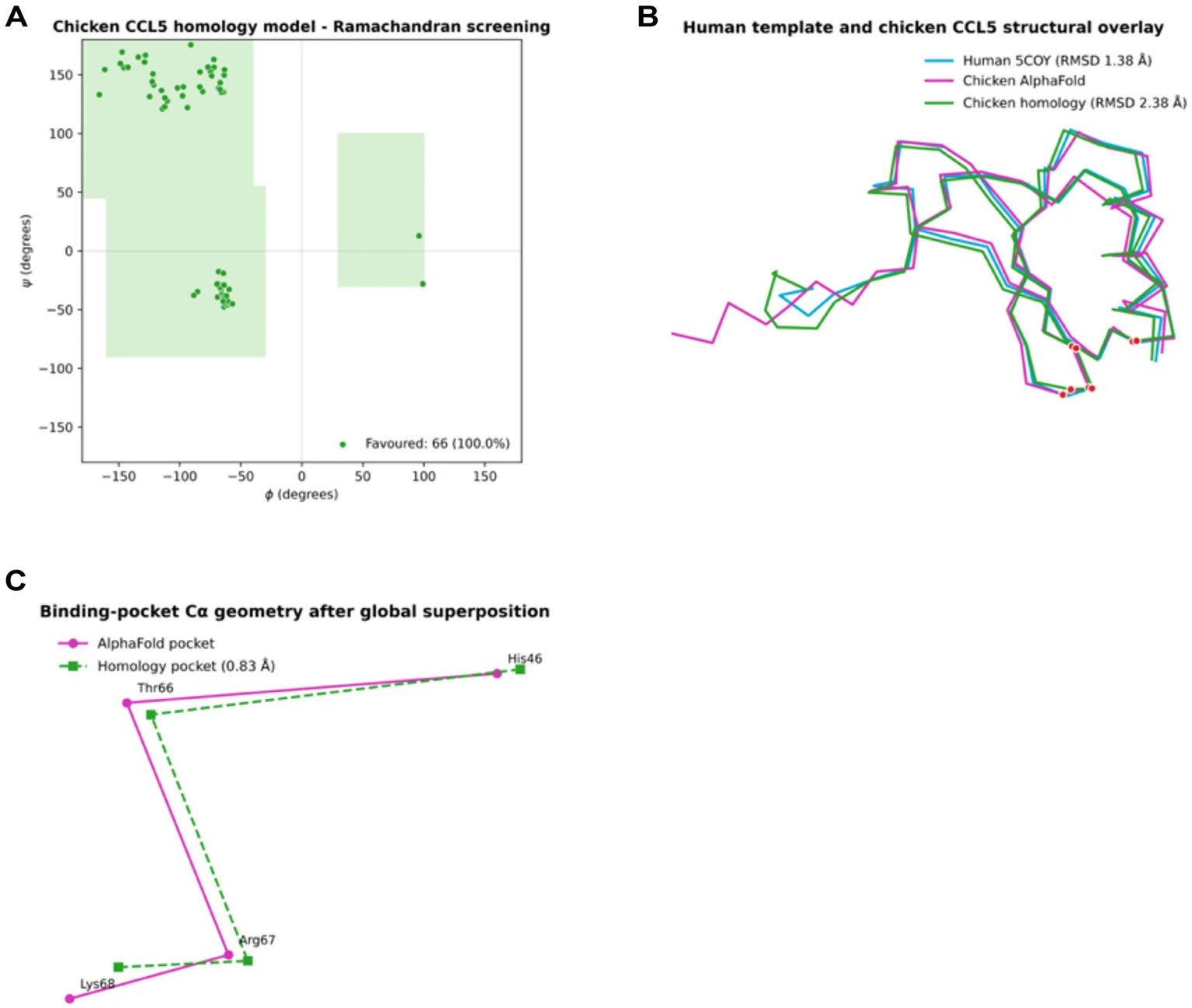
Structural assessment of the chicken CCL5 homology model. **(A)** Internal Ramachandran screening of the selected homology model. **(B)** Superposition of human CCL5 5COY (cyan), chicken AlphaFold CCL5 (magenta), and the chicken homology model (green). **(C)** Comparison of His46, Thr66, Arg67, and Lys68 after global superposition. The four-residue pocket showed a Cα RMSD of 0.83 Å between the AlphaFold and homology models, supporting conservation of the local putative binding-region geometry. The Ramachandran analysis is an internal geometric screen and should not be interpreted as a formal MolProbity or PROCHECK result.

#### Molecular dynamics simulation

The reconstructed CCL5 dimer showed a mean Cα RMSD of 0.256 ± 0.047 nm. In contrast, chain A and chain B showed RMSDs of 0.223 ± 0.046 nm and 0.237 ± 0.041 nm, respectively, indicating relatively consistent chain-level protein behavior throughout the simulation (Table 2). In contrast, the ligand exhibited a larger RMSD after protein fitting (2.588 ± 1.497 nm), indicating substantial displacement relative to the protein frame. The minimum protein–ligand heavy-atom distance averaged 0.753 ± 0.918 nm, while the protein–ligand center-of-mass (COM) distance averaged 2.408 ± 1.195 nm. Although 77.5% of the analyzed frames retained a minimum heavy-atom distance below the 0.45-nm contact threshold, both the minimum heavy-atom distance and COM distance increased markedly after approximately 40 ns. During the ≥40 ns interval, the mean minimum heavy-atom distance increased to 2.230 nm and the mean COM distance to 4.357 nm, indicating substantial loss of close protein–ligand association during the latter part of the trajectory (Figure 8). Thus, although 4-HC showed an initial association with the CCL5 dimer, the progressive increase in protein–ligand distances after approximately 40 ns indicates loss of close ligand association during the later phase of the simulation. The molecular dynamics (MD) trajectory therefore does not provide evidence for persistent ligand confinement or stable binding to CCL5, and the computational findings should be considered hypothesis-generating rather than evidence of a validated CCL5–4-HC interaction.

**Table 2:** Summary of molecular dynamics trajectory re-analysis metrics for the CCL5–4-hydroxycoumarin complex.

Metric	Value
Dimer Cα RMSD	0.256 ± 0.047 nm
Chain A Cα RMSD	0.223 ± 0.046 nm
Chain B Cα RMSD	0.237 ± 0.041 nm
Ligand RMSD after protein fitting	2.588 ± 1.497 nm
Minimum protein–ligand heavy-atom distance	0.753 ± 0.918 nm
Protein–ligand COM distance	2.408 ± 1.195 nm
Frames with <0.45 nm contact	77.5%
Mean minimum distance ≥40 ns	2.230 nm
Mean COM distance ≥40 ns	4.357 nm

Protein Cα RMSD values represent the structural behavior of the PBC-reconstructed CCL5 dimer and its individual chains. Ligand RMSD was calculated after protein fitting to assess ligand displacement relative to the protein. The minimum protein–ligand heavy-atom distance and center-of-mass (COM) distance describe the spatial relationship between 4-hydroxycoumarin and CCL5. A 0.45-nm threshold was used to define close protein–ligand contact. Although 77.5% of analyzed frames retained contacts below this threshold, the marked increase in both minimum heavy-atom distance and COM distance after 40 ns indicates substantial loss of close protein–ligand association during the late stage of the trajectory.

**Figure 8:**
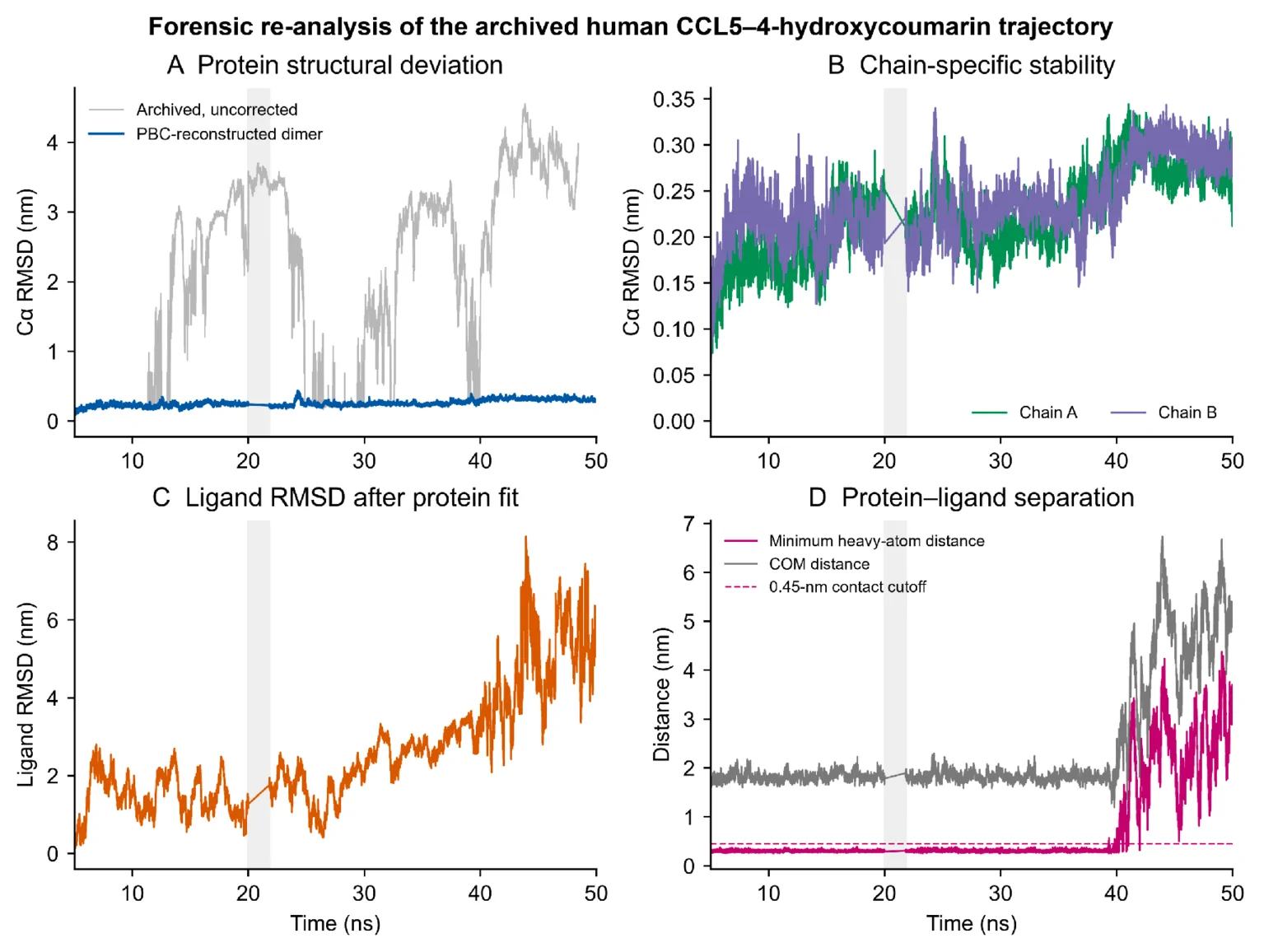
Integrated molecular dynamics analysis of the CCL5-4-hydroxycoumarin complex. **(A)** Protein structural deviation comparing the archived, uncorrected trajectory with the PBC-reconstructed dimer, demonstrating the effect of periodic boundary-condition correction on the apparent protein RMSD. **(B)** Chain-specific protein stability showing the α-carbon RMSD of chains A and B following trajectory correction and protein fitting. **(C)** Ligand RMSD of 4-hydroxycoumarin after protein fitting, illustrating ligand conformational behavior relative to the stabilized protein structure. **(D)** Protein–ligand separation showing the minimum heavy-atom distance and center-of-mass (COM) distance between CCL5 and 4-hydroxycoumarin; the 0.45-nm line denotes the applied contact threshold. The late-stage increase in protein–ligand separation after approximately 40 ns indicates loss of close contact during the latter portion of the trajectory. The trajectory was analyzed over 5–50 ns, with the missing interval from 20.002 to 21.800 ns explicitly retained as a data gap.

#### Clinical manifestations

Infected birds manifested weakness, diarrhea, dehydration, anorexia, and high mortality. Further clinical signs included lowered head, ruffled feathers, drooped wings, and diarrhea. Antibiotic, Ajwain, combination 1 and 2 groups had fewer clinical symptoms when compared to the infected group. No signs of infection were seen in the control group. Post-mortem examination revealed typical lesions, including white raised nodules on the heart, nodular lesions, and cecal cores on the liver, spleen, and intestines in the infected group. Treated groups showed fewer observed pathological findings than the infected untreated group (ANT<COM2 < COM1 < AJWAIN). As each group contained only six birds, the corresponding 95% Wilson confidence intervals were wide. Exploratory two-sided Fisher exact comparisons with INF did not remain statistically significant after Holm correction (all adjusted *p* ≥ 0.303) (Figure 9).

**Figure 9:**
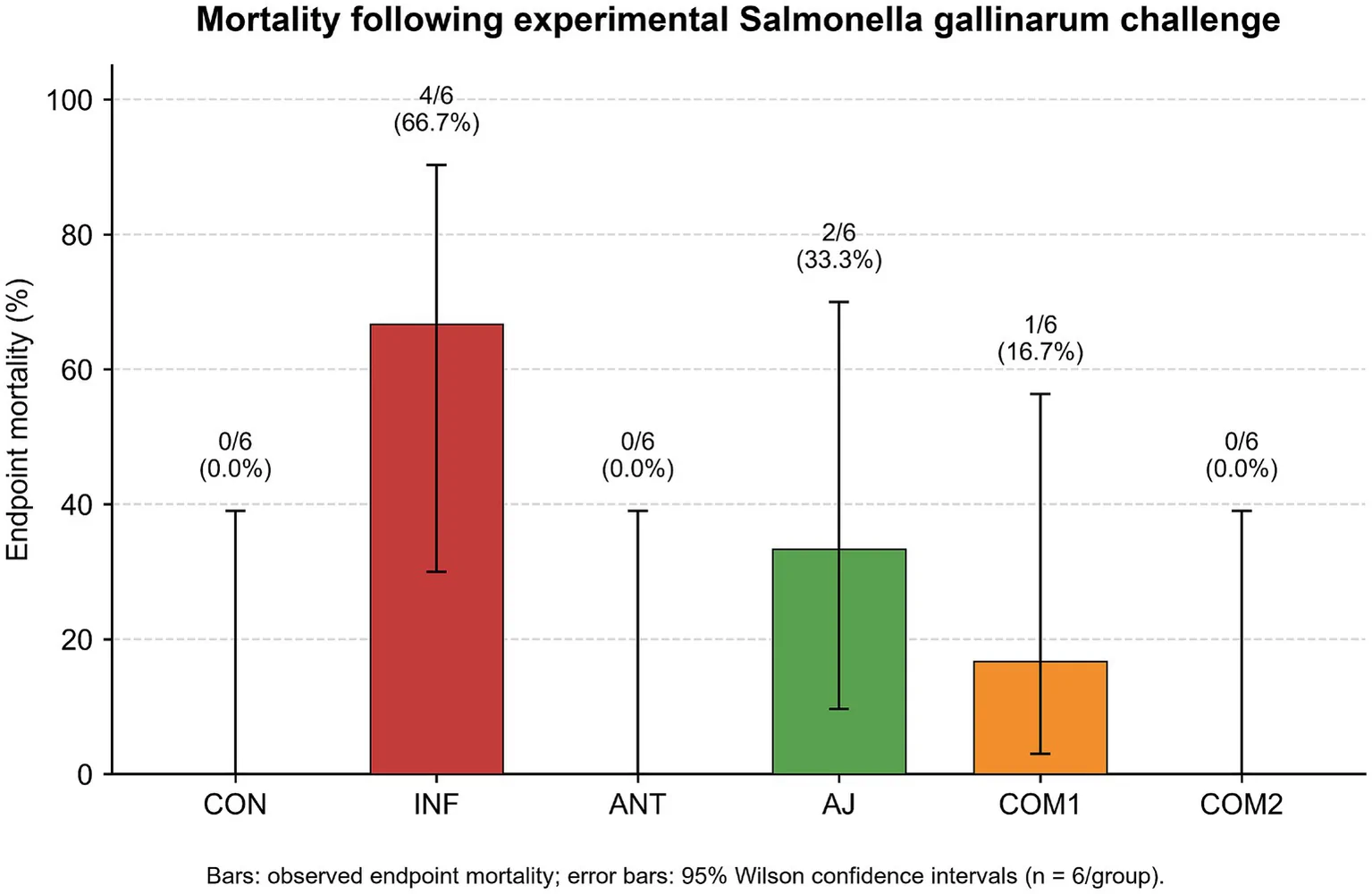
Endpoint mortality by experimental group. Bars show the observed percentage mortality, and error bars show 95% Wilson score confidence intervals. Values above bars indicate deaths/animals at risk. Individual death times were unavailable; therefore, the figure presents endpoint mortality proportions rather than Kaplan–Meier survival estimates. CON, uninfected control; INF, infected untreated; ANT, antibiotic-treated; AJ, Ajwain-treated; COM1, 4-hydroxycoumarin plus silver nanoparticles; COM2, 4-hydroxycoumarin plus silver nanoparticles and Ajwain.

#### Differential gene expression study

Differential gene expression was analyzed using a two-way ANOVA, followed by Tukey’s *post hoc* test to correct for multiple comparisons (Figure 10). Infection with *S. gallinarum* induced a robust pro-inflammatory response, significantly upregulating IL-6, IL-8, IL-12, IL-17A, iNOS, and MMP1 relative to the control (adjusted *p* < 0.0001). All phytocompound treatments significantly attenuated this response compared to the infected group (adjusted *p* < 0.05). The overall expression trend followed CONTROL < ANTIBIOTIC < COMBINATION2 < COMBINATION1 < AJWAIN < INFECTED. Notably, while treatments reduced expression, they did not fully restore baseline levels, as all treated groups remained significantly different from the control (adjusted *p* < 0.05). These findings provide preliminary, descriptive evidence that the tested combination formulations were associated with attenuation of the infection-associated inflammatory response. However, the contribution of individual components, particularly 4-hydroxycoumarin, cannot be independently established from the present *in vivo* design.

**Figure 10:**
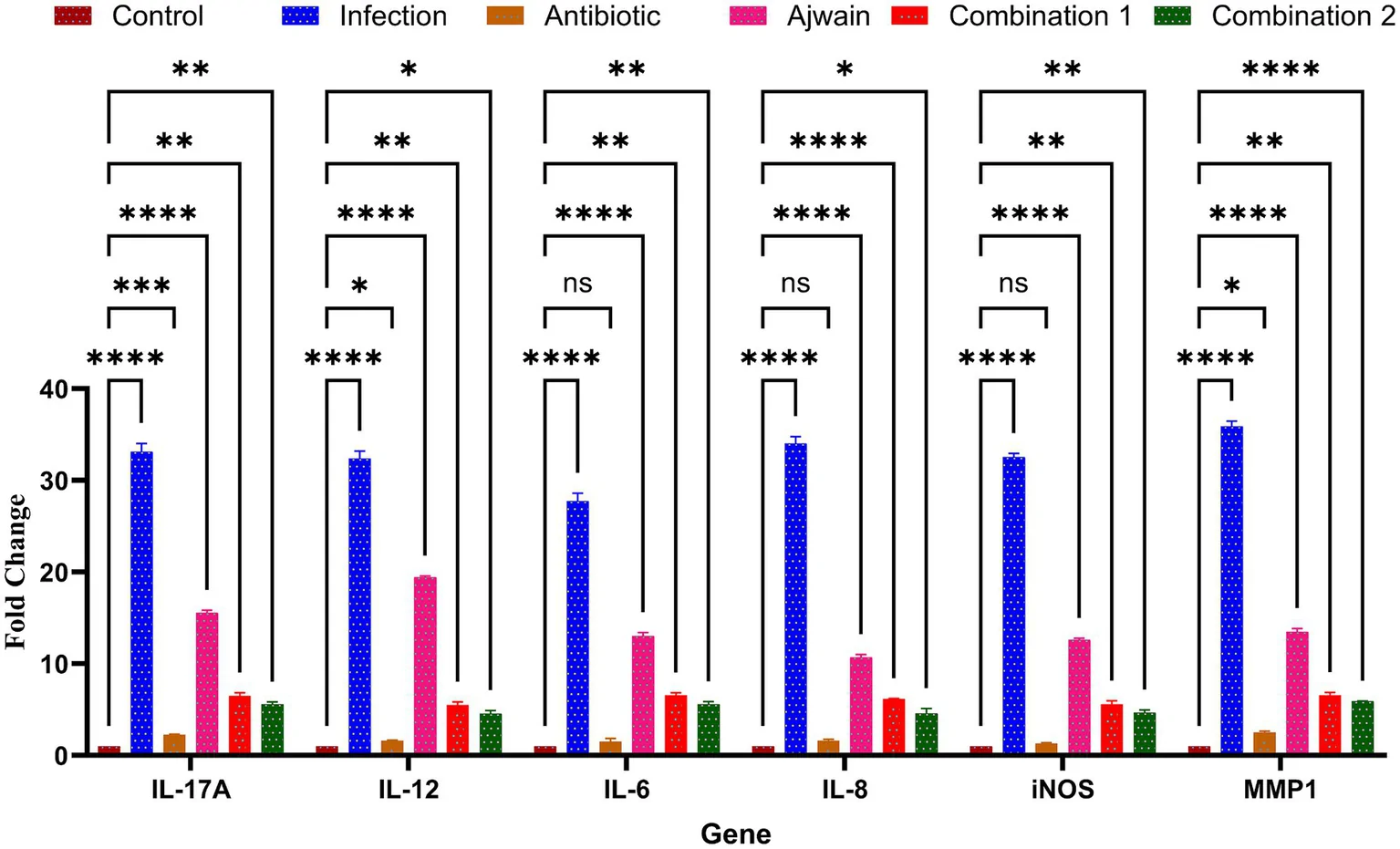
Relative gene expression of inflammatory markers in broiler chicks experimentally infected with *Salmonella gallinarum* and treated with different formulations. The expression levels of six pro-inflammatory genes (IL-17A, IL-12, IL-6, IL-8, iNOS, and MMP1) were measured by qPCR. Data were normalized to the Control group (set to a fold change of 1). Statistical significance was determined using a two-way ANOVA, followed by a Dunnett’s *post hoc* test to correct for multiple comparisons. Brackets indicate statistically significant differences between the indicated groups (* *p* < 0.05, * *p* < 0.01, ** *p* < 0.001, ** *p* < 0.0001; ns = not significant).

## Discussion

*Salmonella enterica* ssp. Enterica Serovar Gallinarum, an avian-restricted pathogen, infects poultry and causes a severe systemic disease called fowl typhoid. It is associated with high mortality rates and affects the poultry industry disproportionately in low- and middle-income countries (Jia et al., 2024). Salmonella transmission occurs through both horizontal and vertical routes, including oral transmission, respiratory transmission, and infection of hens that passes to their offspring (Andino and Hanning, 2015). Various predisposing factors, such as concurrent infection status, pathogen virulence, the bird’s age/breed/species, and flock management, play significant roles in disease-related mortality and morbidity (Alves et al., 2024). Poultry farmers suffer significant losses due to production losses, poor growth rates, and reduced egg production.

The primary treatment regimen for Salmonella is antimicrobial therapy; however, the widespread use of antibiotics has led to major issues. Misuse/overuse of antibiotics leads to antimicrobial resistance and the build-up of antibiotic residues in animal products intended for human consumption (Rodrigues et al., 2020). This necessitates controlling Salmonella infections in poultry and identifying antimicrobial alternatives to curb the indiscriminate use of antibiotics.

Phytocompounds, which are derived from medicinal compounds, are gaining attention in recent times due to their diverse properties, including immunomodulatory, antimicrobial, and anti-inflammatory effects (Riaz et al., 2023). Similar studies have been reported in the literature when plant-derived antimicrobials were used in combinations (Ahmad et al., 2024; Kolbadinejad and Rezaeipour, 2020; Akbari et al., 2016; Haq et al., 2022). These isolated compounds promise to offer an approach to combat pathogens without contributing to the development of antimicrobial resistance otherwise associated with conventional antibiotics. In this study, treated groups showed, descriptively, milder clinical manifestations than the infected, untreated group. This pattern must be interpreted as descriptive & exploratory, although an apparent ordering of improvement was seen across the treatment groups. As between-group mortality comparisons did not remain statistically significant after Holm correction, the present data do not establish a statistically significant ranking of treatment efficacy. In the future, larger, adequately powered studies will be required to determine whether true differences exist among the treatment groups.

Clinical manifestations observed in infected birds were characteristic of *S. gallinarum* infection, indicating systemic illness and severe physiological stress. These findings corroborate with previous reports, which described similar systemic signs resulting from bacterial septicemia and endotoxin release (Ojima et al., 2021). In our study, the combination-treatment groups showed, descriptively, milder clinical manifestations than the untreated infected group. These observations should be interpreted as preliminary, descriptive, and exploratory proof-of-concept findings rather than definitive evidence of therapeutic efficacy. Although an apparent pattern of improvement was observed across the tested groups, the lack of statistical significance after Holm correction prevents definitive attribution of differences in clinical or mortality outcomes to treatment effects. Importantly, these observations apply strictly to the tested treatment combinations and should not be interpreted as evidence for an independent therapeutic effect of 4-hydroxycoumarin.

Coumarins are known for their diverse pharmacological activities, including antimicrobial properties. 4-Hydroxycoumarin and its analogs have gained significant attention in recent years due to their low toxicity profile and broad spectrum of biological activities (Jung and Park, 2009). However, their efficacy in poultry farming has remained relatively unexplored. The innovative approach of using 4-hydroxycoumarin offers potential advantages, including structural diversity, reduced likelihood of resistance, and the ability to modify it to enhance specificity and activity (Gadamsetti et al., 2024). It further enables precise control over their chemical properties through the chemical synthesis of 4-hydroxycoumarin, thereby facilitating optimization of efficacy, safety, and cost-effectiveness.

Computational docking was performed to investigate the potential host-directed anti-inflammatory mechanism of 4-hydroxycoumarin in addition to its direct antibacterial activity. As CCL5 does not possess an experimentally validated small-molecule drug-binding pocket, the cavity identified in the present study should be regarded as a putative ligand-binding site rather than an established druggable pocket. Molecular docking predicted a favorable interaction between 4-hydroxycoumarin and CCL5. To further examine the dynamic behavior of the predicted complex, the archived molecular dynamics trajectory was subjected to an independent forensic re-analysis after correction for periodic-boundary artifacts. The corrected protein trajectory showed relatively consistent chain-level behavior, whereas the ligand displayed substantial displacement relative to the protein frame. Although close protein–ligand contacts were retained in a substantial proportion of analyzed frames, both minimum heavy-atom and center-of-mass distances increased markedly after approximately 40 ns, indicating loss of close association during the latter part of the trajectory. Thus, the MD analysis provides evidence that an initial period of protein–ligand association does not support the previous interpretation of persistent ligand confinement or stable ligand binding. The CCL5 analysis should be interpreted with caution. CCL5 lacks an experimentally validated small-molecule binding pocket, and the cavity identified in this study is a putative site. Furthermore, the MD trajectory did not support persistent ligand association during the latter part of the simulation. Thus, the present computational analysis does not establish direct or sustained binding of 4-HC to CCL5 and instead provides a hypothesis for the future experimental investigation.

The computational findings suggest a possible host-directed mechanism involving CCL5, although the specific *in vivo* contribution of 4-hydroxycoumarin cannot be disentangled from the present combination-treatment design. The biological experiments in the present study were conducted in broiler chicken. Thus, additional structural validation of the avian chemokine was also performed to improve the biological relevance of the computational analyses. The mature chicken CCL5 protein retains a highly conserved chemokine fold relative to its human ortholog, as supported by sequence alignment, structural superposition, and independent homology modeling. Excellent structural agreement between the two proteins suggests that the predicted ligand-binding region is evolutionarily conserved. Nevertheless, molecular docking and molecular dynamics simulations remain predictive computational approaches, and the future experimental studies will be required to validate the interaction between 4-hydroxycoumarin and chicken CCL5.

Previous computational studies have also been done against resistance targets using phytocompounds, underscoring the significant potential of plant-derived compounds as a viable source to combat antimicrobial resistance. A study by Jangid et al. (2025) employed molecular docking and simulation studies to identify phytochemical inhibitors targeting mobilized colistin resistance. Another study screened phytochemicals from medicinal plants for inhibitory potential against a critical resistance protein in MDR *Salmonella typhimurium* (Gaikwad, 2023). CCL5 has been reported to play a central role in the pathogenesis of chronic inflammatory diseases, autoimmune disorders, cancer metastasis, and leukocyte chemotaxis (Zeng et al., 2022; Aldinucci et al., 2020). Thus, the selective interaction of 4-hydroxycoumarin with CCL5, supported by the inherent anti-inflammatory properties, positions it as a promising lead compound for the targeted disruption of CCL5-mediated signaling pathways, necessitating further experimental validation.

Combined formulations target both the pathogen and the host immune response and thus, integrate multiple bioactive components with complementary mechanisms of action to enhance therapeutic efficacy. Such approaches may also reduce the likelihood of antimicrobial resistance and improves bioavailability by permitting the use of lower concentrations of individual components. However, the nature of the interaction among the individual components was not formally established in the present study. As checkerboard assays and fractional inhibitory concentration indices were not determined, the present data cannot establish synergistic interactions among the components. The future studies should therefore use checkerboard assays and FIC-index analysis to distinguish synergistic, additive, antagonistic, and independent interactions.

Previously, the antimicrobial efficacy of Ajwain extract complemented with probiotics exhibited its potential as a promising candidate for further investigation (Haq et al., 2022). The phenolic monoterpenes, mainly thymol and carvacrol, in Ajwain have been widely recognized for disrupting bacterial membranes and inhibiting enzymatic activity (Khwaza and Aderibigbe, 2025). The volatile constituents of Ajwain may facilitate the entry of 4-hydroxycoumarin into bacterial cells, thereby strengthening its antimicrobial action as seen in our combination treatment group.

Silver nanoparticles have been widely investigated as potential carrier systems for antimicrobial compounds because of their large surface area and favorable physicochemical properties (Dakal et al., 2016). In the present study, silver nanoparticles were incorporated into the combination formulation due to their reported antimicrobial activity and potential to enhance formulation performance. The physicochemical characterization of the synthesized AgNPs was limited primarily to morphological assessment by SEM & FTIR. We acknowledge that a more comprehensive physicochemical characterization, including UV–Vis spectroscopy, dynamic light scattering (DLS), zeta-potential analysis, energy-dispersive X-ray spectroscopy (EDX), and X-ray diffraction (XRD), would provide further insight into the optical, colloidal, elemental, and crystallographic properties of the nanoparticles. Furthermore, interactions between 4-hydroxycoumarin and the nanoparticles, including drug loading, adsorption efficiency, release kinetics, and bioavailability, were not experimentally evaluated and therefore warrant further investigation to enhance the stability and solubility of hydroxycoumarin and enable efficient cellular uptake. Recent advances in Ag-based nanocomposite systems further support the potential of silver-containing formulations as antimicrobial platforms. For example, Ag@HKUST-1 nanocomposites encapsulating *Cinnamomum sintoc* essential oil have demonstrated enhanced antibacterial activity against *Salmonella Typhi,* highlighting how combining silver nanomaterials with bioactive phytochemicals can improve antimicrobial performance (Beladona et al., 2026). Likewise, recent studies on bio-derived silver nanomaterials and functional nanostructures have emphasized the importance of integrating phytochemicals with nanomaterials to enhance stability, antimicrobial efficacy, and biocompatibility (Rosmainar et al., 2026). Although the present study did not investigate nanoparticle–drug interactions or loading efficiency, our findings are consistent with the growing body of literature supporting phytochemical–silver nanoparticle combinations as promising alternatives to conventional antibiotics. Previously, polyvinylpyrrolidone-Capped silver nanoparticles have been used as antimicrobial agents against diverse microbes (Tiwari et al., 2017). Overall, incorporation of silver nanoparticles may have contributed to the therapeutic performance of the combination formulation, although dose optimization, AgNP biodistribution and tissue accumulation, pharmacokinetic profiling, and dedicated short- and long-term toxicological studies are required to establish the safety margin and translational suitability of this formulation.

The combined formulation showed high cell viability in the chicken fibroblast assay at the tested concentrations, indicating low cytotoxicity under the conditions evaluated. However, this *in-vitro* observation should not be interpreted as evidence of systemic safety in poultry. In particular, the present study did not assess AgNP biodistribution, tissue accumulation, pharmacokinetics, or dose-dependent toxicity. Therefore, dedicated *in-vivo* safety studies will be required before the formulation can be considered for translational or field application.

The descriptively lower clinical severity, gross lesion burden, and endpoint mortality observed in the treated groups represent preliminary and exploratory observations that require confirmation in larger, adequately powered studies (Abd El-Hack et al., 2022). To evaluate the anti-inflammatory potential of 4-hydroxycoumarin-based formulations, the relative mRNA-based expression of key pro- and anti- inflammatory cytokines such as IL-6, IL-8, IL-17A, IL-12, and iNOS was quantified using qRT-PCR. A marked decrease in cytokine expression was observed with 4-hydroxycoumarin, particularly when administered in combination with Ajwain extract and silver nanoparticles. The reduction suggests that the combined formulation effectively modulated the host inflammatory response. In contrast, groups treated with 4-hydroxycoumarin with nanoparticles exhibited moderate suppression, suggesting a potentially greater modulatory effect of the combined formulation under the conditions evaluated.

Collectively, these findings underscore the therapeutic potential of this approach as part of a natural, multi-component intervention strategy for controlling bacterial infections in poultry. Although the present results are encouraging, larger field-based trials, pharmacokinetic profiling, and residue analyses are warranted to confirm long-term safety and commercial viability. Future investigations should also explore the molecular pathways involved in host immune modulation and evaluate the efficacy of this formulation against other multidrug-resistant pathogens affecting poultry.

A limitation of the present study is that 4-hydroxycoumarin was not evaluated as a standalone treatment in the *in-vivo* model. Therefore, the contribution of 4-HC to the observed effects cannot be independently established. The *in-vivo* findings should be interpreted strictly in relation to the tested treatment combinations and should not be considered evidence of an isolated therapeutic effect of 4-HC. Although the combination formulations were associated with improved clinical, pathological, and inflammatory outcomes, the individual contribution of 4-hydroxycoumarin could not be independently assessed. The future studies should include a 4-hydroxycoumarin-only treatment group to delineate its specific *in vivo* effects and better characterize the contribution of each component within the formulation. MIC and MBC assays of the individual test agents were performed against *S. gallinarum* to evaluate their antibacterial activity. All test agents demonstrated antibacterial activity, although differences in inhibitory concentrations were observed among the individual compounds. The future studies should employ checkerboard assays to determine the fractional inhibitory concentration (FIC) as well as quantitatively evaluate the efficacy of the individual compounds and the combination formulation.

An important limitation of the present study is the small experimental group size of six birds per group and the absence of a prospective statistical power calculation. The resulting limited sample size may have reduced the ability to detect between-group differences, particularly for mortality outcomes. Accordingly, the observed differences among treatment groups represent preliminary, descriptive, and exploratory proof-of-concept findings and should not be considered definitive evidence of differential treatment efficacy.

Before commercial implementation, additional studies are needed to evaluate long-term safety, pharmacokinetics, residue profiles, large-scale field efficacy under commercial farming conditions, and effectiveness against multidrug-resistant clinical isolates. Therefore, the present work should be regarded as a proof of concept that establishes a strong foundation for future translational research and technology transfer to the poultry industry.

## Conclusion

Our findings provide proof of concept for a multi-component phytochemical–nanotechnology strategy that integrates direct antimicrobial activity with modulation of the host inflammatory response during fowl typhoid. Our findings provide preliminary, descriptive, and exploratory proof-of-concept evidence for a multi-component phytochemical–nanotechnology approach that integrates direct antimicrobial activity with modulation of the host inflammatory response during fowl typhoid. The observed *in vivo* effects belong strictly to the tested treatment combinations containing 4-hydroxycoumarin, Ajwain extract, and/or silver nanoparticles and should not be interpreted as definitive therapeutic validation. In particular, 4-hydroxycoumarin was not evaluated as a standalone treatment *in vivo*; therefore, its individual contribution to the observed outcomes remains unproven. Furthermore, comparisons of mortality and clinical endpoints did not remain statistically significant after Holm correction and should therefore be interpreted descriptively and exploratorily. The computational analyses provide hypothesis-generating evidence for a possible interaction between 4-hydroxycoumarin and CCL5, but the predicted binding site and dynamic association require experimental validation. Larger, adequately powered *in vivo* studies incorporating standalone component groups and comprehensive nanoparticle characterization will be required to establish treatment-specific efficacy and safety.

## Data Availability

The original contributions presented in the study are included in the article/Supplementary material, further inquiries can be directed to the corresponding authors.
