# Supplementary material for: A phytochemical–nanotechnology platform exhibits complementary antimicrobial and immunomodulatory effects in experimental fowl typhoid
Source: Front Microbiol. 2026 Oct 1;17:1894409. doi: 10.3389/fmicb.2026.1894409 (PMC13668857; doi:10.3389/fmicb.2026.1894409)
Supplement: Supplementary file 1 [file Supplementary_file_1.DOCX]

**Table 1. Primer sequences used for quantitative real-time PCR (qRT-PCR) analysis.** The table lists the target genes analyzed in this study, along with their corresponding forward primer (FP) and reverse primer (RP) sequences, expected amplicon size (bp), and optimized annealing temperature (TA, °C) used for amplification. All primers were synthesized commercially and used for the quantification of gene expression in experimental broiler chick tissue samples.

| Target | Primer Sequence 5’-3’ | T_A_°C | Amplicon | References |
| --- | --- | --- | --- | --- |
| β – Actin | FP (TGGCATTGCTGACAGGAT)  RP (CTGCTTGCTGATCCACAT) | **63** | **160 bp** | (X. M. He et al., 2013) |
| IL-6 | FP (GCTCGCCGGCTTCGA)  RP (GGTAGGTCTGAAAGGCGAACAG) | **63** | **71 bp** | (Van Goor et al., 2016) |
| IL-17A | FP (TTTCTGCACATGGGAAGGTG  RP (CCTGGTTCATGTTGCTGATGA) | **61** | **144 bp** | (S. H. Lee et al., 2014) |
| IL-8 | FP (GGCTTGCTAGGGAAATGA)  RP (AGCTGACTCTGACTAGGAAACTGT) | **55** | **200 bp** | (Khampeerathuch et al., 2018) |
| iNOS | FP (AAAGAAAGGGATCAAAGGTGGT)  RP (CAAGCATCCTCTTCAAAGTCTG) | **60** | **296 bp** | (Palamidi et al., 2016) |
| IL-12 | FP (TGGTCCACGCTTTGCAGAT)  RP (AAGGTTAAGGCGTGGCTTCTTA) | **65** | **111bp** | (Berndt et al., 2007) |
| MMP1 | FP(ATGCTTTTCAGCCAGGGAA)  RP(CTTTGGGGTCTGTGTAGGCA) | **60** | **192** | (Yin et al., 2016) |

**Table 2.** Antibacterial activity of 4-hydroxycoumarin, Ajwain (*Trachyspermum ammi*) extract, and silver nanoparticles against *Salmonella gallinarum* determined using the disc diffusion assay. Antibacterial activity is expressed as the diameter of the inhibition zone (mm). Concentrations are presented as the final concentration of each test compound. Values represent the mean of three independent experiments performed in triplicate. Ciprofloxacin served as the positive control, while the respective solvent served as the negative control.

| **Compound** | **Concentration (µg/mL)** | **Zone of Inhibition (mm)** |
| --- | --- | --- |
| **4-Hydroxycoumarin** | 200 | 4 |
|  | 400 | 8 |
|  | 600 | 13 |
|  | 800 | 19 |
| **Ajwain extract** | 600 | 6 |
|  | 900 | 8 |
|  | 1200 | 13 |
| **Silver nanoparticles** | 25 | 6 |
|  | 50 | 8 |
|  | 100 | 10 |

**Table 3.** Antibacterial activity of different combinations of 4-hydroxycoumarin, Ajwain (Trachyspermum ammi) extract, and silver nanoparticles against Salmonella gallinarum determined using the disc diffusion assay. The amount of each component is expressed as **µg per well**, and antibacterial activity is presented as the diameter of the inhibition zone (mm). COM-A, 4-hydroxycoumarin + Ajwain extract; COM-B, 4-hydroxycoumarin + silver nanoparticles; COM-C and COM-D, ternary combinations of 4-hydroxycoumarin, Ajwain extract, and silver nanoparticles at different doses. Values represent the mean of three independent experiments performed in triplicate. Ciprofloxacin served as the positive control and the respective solvent served as the negative control.

| **Combination** | **Composition** | **Final Amount (µg/well)** | **Zone of Inhibition (mm)** |
| --- | --- | --- | --- |
| **COM-A** | 4-Hydroxycoumarin + Ajwain extract | 200 + 300 | 10 |
| **COM-B** | 4-Hydroxycoumarin + Silver nanoparticles | 200 + 10 | 11 |
| **COM-C** | 4-Hydroxycoumarin + Ajwain extract + Silver nanoparticles | 200 + 150 + 5 | 16 |
| **COM-D** | 4-Hydroxycoumarin + Ajwain extract + Silver nanoparticles | 200 + 300 + 10 | 17 |

**Ciprofloxacin disk zone of inhibition = 28mm**

**Figure 1. Antibacterial activity of selected compounds against Salmonella gallinarum determined by the disk diffusion assay.** Representative agar plates showing the zones of inhibition produced by 4-hydroxycoumarin, Ajwain (Trachyspermum ammi) extract, silver nanoparticles, and their selected combinations against Salmonella gallinarum. The diameter of the clear inhibition zone surrounding each disk reflects the antibacterial activity of the respective treatment. Ciprofloxacin served as the positive control, while the respective solvent served as the negative control.
